# The Association of Body Mass Index With Mortality Among Pulmonary Hypertension Patients: A Systematic Review and Meta-Analysis of Cohort Studies

**DOI:** 10.3389/fpubh.2022.761904

**Published:** 2022-05-10

**Authors:** Chaoxin Jiang, Xiongde Fang, Wenjin Fu

**Affiliations:** ^1^Department of Laboratory, Guangdong Provincial Hospital of Integrated Traditional Chinese and Western Medicine, Foshan, China; ^2^Department of Pathology, Guangzhou Chest Hospital, Guangzhou, China; ^3^Department of Laboratory, Affiliated Houjie Hospital, Guangdong Medical College, Dongguan, China

**Keywords:** body mass index, mortality, pulmonary hypertension, meta-analysis, dose-response

## Abstract

**Objective:**

To run a systematic review and meta-analysis of related studies on body mass index (BMI) and the risk of death among pulmonary hypertension (PH) patients, as well as, to shed light on the shape and strength of the dose-response association.

**Methods:**

Studies published up to Jun 2021 in scientific databases such as Scopus, and PubMed as well as Google Scholar were searched. Cohort studies that reported risk estimates for at least two categories of BMI or per certain increase in BMI in relation to mortality in PH patients were included. Summary relative risks were determined with random effects models. Non-linear relationship was discovered with dose-response analysis.

**Results:**

All in all, 15 cohort studies were selected. The number of participants was 127,215 out of which 73,999 were reported dead. The summary RR for mortality per a 5-unit increment in BMI was 0.83 (95% confidence interval 0.77–0.89; *I*^2^ = 75.6%, *n* = 9) among PH patients. There was a non-linear dose-response relation between BMI and mortality in PH patients (P_non−linearity_ < 0.001), with the lowest risk being at BMI 32–38 kg/m^2^.

**Conclusion:**

Higher BMI is related to decreased risk of mortality among PH patients and the lowest point of the curve was seen at BMI 32–38.

## Introduction

Obesity is considered as one of the main contributors to health issues throughout the world (1, 2). Body Mass Index (BMI) has regularly been used to measure the amount of excess fat in human body. BMI has been approved by the World Health Organization (WHO) as a reliable means of measuring obesity (3) despite the fact that it does not measure the distribution of fat in one's body directly. A bulk of research have shown that obesity can independently be a cardiovascular risk factor that significantly increases the chances of coronary artery disease, pulmonary hypertension (PH), disordered breathing, heart failure (HF), and increased morbidity and mortality (4–7).

Pulmonary arterial hypertension (PAH) is an infrequent cardiopulmonary disease that only 15–150 people per million experience it (8). Age-standardized mortality rates related to PH has been reported between 4.5 and 7.3% in African Americans and 5–5.5% in whites (9). In addition, the survival rates of PH at 1, 3, and 5 years have been indicated to be 68, 48, and 34%, respectively, with an estimated median survival of 2.8 years (10).

Being a consequence of pathophysiological changes in pulmonary vascular structure, which often leads to unusually high pulmonary artery pressures, pulmonary arterial hypertension (PAH), is known as a key cardiovascular condition which typically results in severe cases such as right-sided heart failure as well as death (4). PAH patients suffer from higher catabolic burden and cachexia due to higher baseline inflammatory condition (11). Thus, additional adipose tissue may have beneficial effect in PAH patients.

Recent data showed that an independent positive relationship between obesity and PH (7, 12). BMI has been confirmed to be conversely related to long term mortality in hemodialysis patients as well as some chronic diseases, such as chronic obstructive pulmonary disease, peripheral vascular disease, and coronary artery diseases (5, 13–16).

Although obesity is considered as an important independent PH risk factor, the existing data show decreased mortality in obese PH patients (17–20). The term “obesity paradox” has been coined for this epidemiological finding.

Considering the lack of systematic review and meta-analysis to examine the relation of BMI with mortality in PH patients, this study aimed to evaluate such associations.

## Materials and Methods

The framework of the present study was based on the principles of the Preferred Reporting Items for Systematic Reviews and Meta-Analyses (PRISMA) statement (21).

### Search Strategy

PubMed, Scopus, and Google Scholar were explored using relevant terms to find related research papers published up to Jun 2021. The selected keywords were: [(“body mass index” OR BMI OR “Obesity” OR “Obesity/complications” OR “overweight” OR “adiposity”) AND (survival OR mortality OR death OR “Survival Rate”) AND (“Pulmonary Hypertension”)]. No filters were applied for searching the above-mentioned databases. In order to prevent any missing or unknown article, the reference lists of the included articles and reviews were manually inspected.

### Inclusion Criteria

Two investigators evaluated the titles, abstracts, and, in some cases, the full texts of papers, taking into considerations the following inclusion criteria: (1) cohort studies, (2) studies on the association of BMI with risk of mortality, (3) studies conducted on PH patients, (4) those reported hazard ratio (HR), rate ratio (RR), and odds ratio (OR) and the corresponding 95% confidence interval (CI) in a linear or categorical approach in discovering the associations (5) studies with English language. When encountering paradoxes, the investigators managed to find solutions by discussing them with the Principal Investigator.

Studies which were concerned with patients other than PH sufferers or those which concentrated on other types of hypertensions, such as systemic blood pressure along with PH were excluded. In the same vein, studies with similar populations, and studies that assessed obesity based on ICD-9 codes and did not provide enough information on body mass index, were also removed. Last but not least, review articles, randomized clinical trials, abstracts, unpublished studies, letters and comments, as well as *in vitro* or animal studies were not included in this study.

### Data Extraction

Each paper was carefully examined by two independent authors and the following information was extracted: author's first name, country, study design, participants' age mean/range, publication year, PH and mortality assessment approach, duration of follow-up, the number of PH patients and deaths, used cut-points for PH definition, participants' gender, continuous or categorical values, and adjustment. We extracted the adjusted RRs when the studies had both crude and adjusted RRs. In cases where the two authors did not agree on an issue, the corresponding author mediated in resolving the point of disagreement.

### Risk of Bias Assessment

In order to measure risk of bias, with respect to each eligible study the Newcastle-Ottawa Scale (NOS) scale (22) was used. This scale comprises three parts, namely; selection, comparability, and exposure or outcome. The total score ranged between 0 and 9. In the present study, those research papers which had a score of 7 or above met our high-quality criteria. Meanwhile, the two authors evaluated each study separately, and if there was a discrepancy, a final decision was made after consulting the PI.

### Statistical Analysis

The random-effects model was applied to determine estimated risk with 95% CIs to combine the RRs of five-increment in BMI and mortality in PH patients. Heterogeneity among included studies was assessed using *I*^2^ test. The *I*^2^ values of 25–50, 50–75, and >75% were defined as low, moderate, and high heterogeneity, respectively (23). Subgroup analysis and meta-regression were also run to detect the sources of heterogeneity focusing on the following variables: confounder adjustments (age, smoking), mediator adjustment (diabetes, hypertension), country, sample size, quality score, and duration of follow-up. In order to detect any evidence of publication bias, Funnel plot and egger test was applied (24). Also, Sensitivity analysis was performed to assess the impact that each study had on summary effect size.

In addition, dose-response analyses was conducted using the methods suggested by Greenland and Longnecker (25) and Orsini et al. (26). A two-stage random effects dose-response meta-analysis was carried out to examine possible non-linear associations between BMI and mortality in PH patients (26). The number of deaths, person-year, BMI range, and risk estimates for each category were extracted from studies which had at least two BMI categories. For those studies which did not report the number of deaths or PH patients in each category, our presupposition was that it was similar across all categories. Non-linear associations were investigated by modeling exposure levels with the using limited cubic splines with 3 knots at the 10th, 50th, and 95th percentiles of the distribution (27). The null hypothesis for this study was as follows: coefficient of the second spline is equal to zero.

Also, a linear dose-response association between BMI and risk of death in PH patients was identified and examined for each 5 kg/m^2^ augment in BMI by using a two-stage generalized least-squares trend estimation method (25, 26, 28). First, study-specific slope lines were calculated then, these lines were merged so that an overall average slope could be obtained (26). Study-specific slope lines were combined applying a random effects model (29). STATA software version 16.0 was used in all statistical analyses. The *P*-value below 0.05 was considered as significant.

## Results

**Figure 1:**
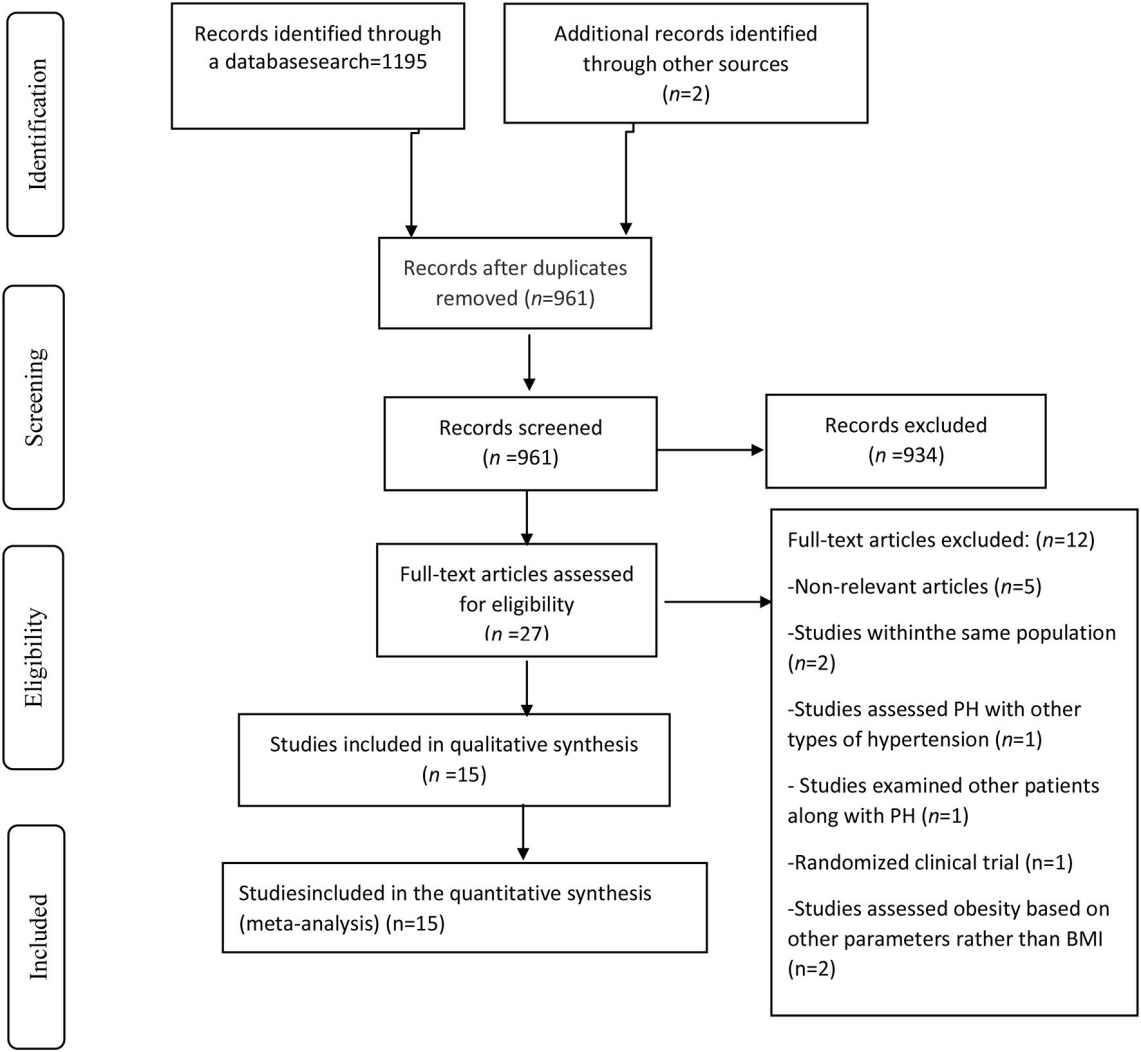
Study selection flow diagram.

Going through a systematic literature search we obtained 1,197 articles after removing duplicates (Figure 1). After reviewing the articles' titles and abstracts, a total of 934 publications were decided to be excluded. Of the 27 remaining publications, 12 papers were excluded because: They were either an RCT study or a review article; PH and systemic hypertension were simultaneously analyzed; the same population was chosen for more than one study; participants other than PH patients were included in the study; Obesity was assessed with parameters other than BMI, or the presented results were irrelevant. On the whole, 15 cohort studies met our criteria and were selected for the final systematic review and meta-analysis (7, 12, 17–20, 30–38).

As mentioned above, a total of fifteen studies were selected which, all in all, reported on a total of 127,215 participants and 73,999 incident cases published from 2005 to 2020. Most of relevant studies were conducted in US (*n* = 8) (7, 12, 18, 19, 30, 32, 35, 37), while others were run in China (*n* = 2) (20, 31), Kenia (*n* = 1) (34), France (*n* = 1) (38), Italy (*n* = 1) (33), Israel (*n* = 1) (17), Australia and New Zealand (*n* = 1) (36). All studies focused on risk estimates for males and females. The follow-up duration in the cohort studies varied from 0.5 to 8 years. Also, participants' age range varied between 18 and 80 years old. Table 1 depicts a more detailed description of different features of the included studies. In most of these publications, important cofounders such as age (*n* = 7), smoking (*n* = 2), and mediators including diabetes (*n* = 6), hypertension (*n* = 3) were adjusted in the analysis. Among the selected essays, six of them enjoy a rather high methodological quality (score ≥7) (7, 18, 20, 34, 37, 38), while the other nine have a low quality (<7) (Table 2).

**Table 1:** Characteristics of cohort studies eligible in the systematic review and meta-analysis.

**Code**	**References**	**Country**	**Study setting**	**Sample Size/death (gender)**	**Age (yrs)**	**Follow-up period**	**Exposure**	**BMI assessment**	**Median/ cutoff point**	**RR (95%CI)**	**PH definition**	**Adjustments**
1	Frank et al. (7)	US	Patients undergoing clinically indicated right-sided heart catheterization	5,453/321 (male and female)	18–80	5.5 yrs	Obesity	Baseline	BMI <30 BMI ≥ 30	1 0.77 (0.69–0.85)	mPAP > 20 mmHg	Age, sex, heart rate, hypertension, diabetes mellitus, obstructive sleep apnea, chronic kidney disease, previous myocardial infarction, and heart failure
2	Ngunga et al. (34)	Kenia	Patients diagnosed with moderate to severe PH at Aga Khan University Hospital	659/198 (male and female)	65.72 ± 17.45	626 d	Obesity	Baseline	BMI <30 BMI ≥ 30	1 0.66 (0.48–0.90)	PASP > 45 mmHg	Age, gender, race and presence of diabetes mellitus
3	Min et al. (19)	US	Patients with newly diagnosed or established PAH who enrolled in the PHAR at one of the fifty participating Pulmonary HypertensionCare Centers.	767/94 (male and female)	≥18	527 d	Obesity	Baseline	BMI: 18.5–24.9 BMI: 25–29.9 BMI ≥ 30	1 0.48 (0.25–0.89) 0.43 (0.23–0.78)	NR	Age, sex, race/ethnicity, etiology, cardiac index, right atrial pressure, receiving combination PAH-therapy, use of a parenteral prostacyclin analog, use of supplemental oxygen, and referral to lung transplantation
4	Trammell et al. (37)	US	Veterans receiving medical care in the Veterans Health Administration (VHA) system and diagnosed with PH	110,495/ 71,045 (male and female)	70.2	6 mo	Obesity	Baseline	BMI <18.5 BMI: 18.5–24.9 BMI: 25–29.9 BMI ≥ 30	1.73 (1.66–1.81) 1 0.71 (0.70–0.72) 0.56 (0.55–0.57)	ICD-9	Age, gender, race, PH type, diabetes, and pre-PH weight trend
5	Weatherald et al. (38)	France	Patients with idiopathic, drug-induced, and heritable PAH from the French Pulmonary Hypertension Network registry	1,255/379 (male and female)	≥18	5 years	Obesity BMI	Baseline	BMI <18.5 BMI: 18.5–24.9 BMI:25–29.9 BMI: 30–35 BMI ≥ 35 Per 1 kg/m^2^ increase in BMI	1.76 (0.97–3.19) 1 0.85 (0.64–1.12) 0.98 (0.71–1.36) 1.42 (0.95–2.14) 1.01 (0.99–1.03)	mPAP > 25 mmHg, PAWP ≤ 15 mmHg, PVR > 3 wood units	Age, sex, etiology of pulmonary arterial hypertension, systemic hypertension, diabetes, smoking, New York Heart Association functional class, right atrial pressure, mean pulmonary arterial pressure, and cardiac index
6	Yang et al. (12)	US	Adult patients referred for RHC in both inpatient and outpatient settings	4,576/1,720 (male and female)	NR	4.7 years	BMI	Baseline	An increment from the 25th to 75th percentile value	0.75 (0.66–0.85)	mPAP > 25 mmHg	NR
7	Strange et al. (36)	Australia and New Zealand	Prospective cohort	220/40 (male and female)	57.2 ±18.7	26 mo	Obesity	Baseline	BMI <30 BMI ≥ 30	1 0.91 (0.85–0.97)	mPAP > 25 mmHg, PAWP ≤ 15 mmHg	Sex and six-minute walk distance
8	Mazimba et al. (18)	US	PHSANZ Registry collects data from patients with all subgroups of PH	267/NR (male and female)	30-50	5 years	BMI	Baseline	Per 1 kg/m^2^ increase in BMI	0.66 (0.52–0.77)	mPAP > 25 mmHg	Age, gender,PH connection risk equation
9	Marini et al. (33)	Italy	Scleroderma subjects were referred center for hemodynamic and respiratory evaluation (SSc-PAH)	49/17 (male and female)	62	48 mo	Obesity	Baseline		1 0.88 (0.78–1)	NR	NR
10	Hu et al. (31)	China	Patients in whom IPAH was diagnosed inFuwai Hospital	173/57 (male and female)	14-59	31.2 mo	BMI	Baseline	Per 3.65 kg/m^2^ increase in BMI	0.53 (0.37–0.74)	mPAP > 25 mmHg	NR
11	Poms et al. (35)	US	Patients with PH diagnosed at participating institutions were enrolled in the Reveal registry.	2959/NR	≥19	3 years	Obesity	Baseline	BMI <30 BMI ≥ 30	0.73 (0.61–0.86)	(PCWP) or left ventricular end-diastolic pressure ≤ 15 mm Hg at diagnosis	Hypertension, Type II diabetes, COPD, sleep apnea, clinical depression, Thyroid disease
12	Zafrir et al. (17)	Israel	PH patients, who underwent echocardiographic and hemodynamic evaluation at PH referral tertiary medical center	105/30 (male and female)	66 ± 12	19 ± 13 mo	Obesity	Baseline	BMI <30 BMI ≥ 30	1 0.2 (0.1–0.6)	mPAP > 25 mmHg	Age, gender, smoking, diabetes mellitus and heartfailure measures
13	Zeng et al. (20)	China	Adult patients who received a diagnosis of IPAH at Fu Wai Hospital	77/32 (male and female)	32	16 mo	BMI	Baseline	Per 3.3 kg/m^2^ increase in BMI	0.51 (0.29–0.91)	mPAP > 25 mmHg	NR
14	Campo et al. (30)	US	Patients with SSc were diagnosed with PAH by heart catheterization in a single center	76/42 (male and female)	61 ± 11	36 mo	BMI	Baseline	Per 1 kg/m^2^ increase in BMI	1.01 (0.95–1.06)	mPAP > 25 mmHg, PCWP ≤ 15 mmHg	No
14	Kawut et al. (32)	US	All patients assessed for primary or secondary pulmonary hypertension who were assessed by clinicians at our center.	84/24	42 ± 14	3 years	BMI	Baseline	Per 1 kg/m^2^ increase in BMI	0.96 (0.90–1.00)	ICD-9	No

*BMI, Body mass index; CAD, chronic artery disease; CKD, Chronic Kidney patients; COPD, Chronic obstructive pulmonary disease; DPG, diastolic pressure gradient; ICD-9, International Classification of Diseases-9; mPAP, mean pulmonary arterial pressure; NR, not reported; PAWP, pulmonary arterial wedge pressure; PCWP, pulmonary capillary wedge pressure; PH, pulmonary hypertension; PVR, pulmonary vascular resistance; RR, relative risk; US, United States*.

**Table 2:** Quality assessment of cohort studies based on Newcastle-Ottawa Scale.

**References**	**Representativeness of the exposed cohort**	**Selection of the non-exposed cohort**	**Ascertainment of exposure**	**Outcome of interest was not present at the start of the study**	**Age adjustment**	**Controls for any additional factor**	**Assessment of outcome**	**Follow-up long enough**	**Adequacy of follow-up of cohorts**	**Total**
Zafrir et al. (17)	*	*		*	*	*	*			6
Frank et al. (7)	*	*	*	*	*	*	*		*	8
Ngunga et al. (34)	*	*	*	*	*	*	*		*	8
Min et al. (19)	*	*		*	*	*			*	6
Marini et al. (33)	*	*		*		*	*		*	6
Strange et al. (36)	*	*		*		*	*		*	6
Trammell et al. (37)	*	*		*	*	*	*		*	7
Weatherald et al. (38)	*	*		*	*	*	*		*	7
Hu et al. (31)	*	*	*	*		*			*	6
Campo et al. (30)	*	*		*			*		*	5
Zeng et al. (20)	*	*	*	*		*	*		*	7
Yang et al. (12)	*	*		*		*	*		*	6
Mazimba et al. (18)	*	*		*	*	*	*		*	7
Kawut et al. (32)	*	*		*			*			4
Poms et al. (35)	*	*		*		*			*	5

### Meta-Analysis

**Figure 2:**
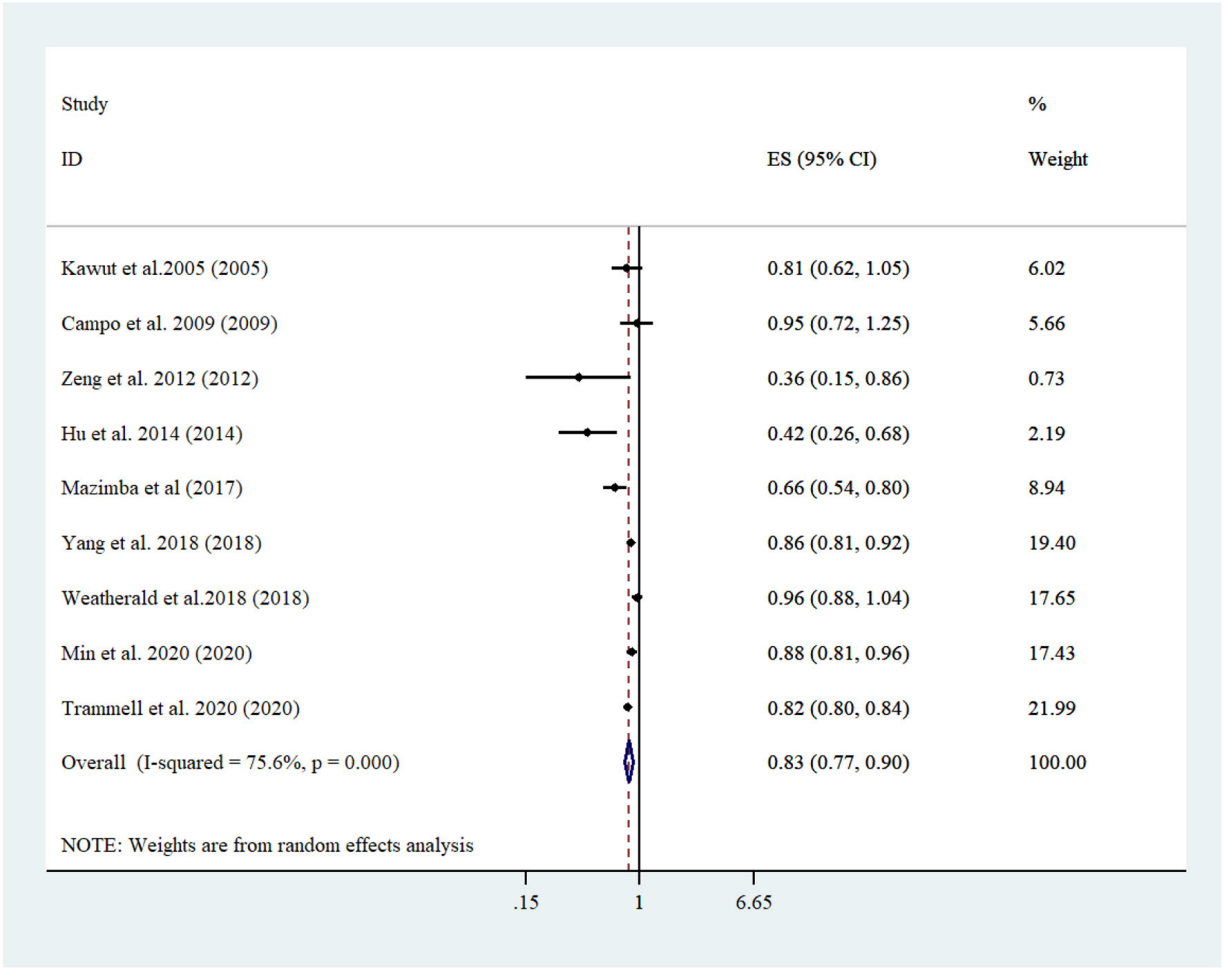
Forest plot derived from random-effects meta-analysis of studies investigating the association between 5-unit increment in body mass index and mortality among pulmonary hypertension patients. CI, confidence interval; ES, effect size.

A linear trend estimation revealed that a 5 kg/m^2^ increment in BMI was related to 17% lowered risk of mortality among PH patients [Pooled risk estimate: 0.83 (95% CI: 0.77, 0.89; *I*^2^ = 75.6, *n* = 9) (Figure 2)]. No evidence of publication bias was confirmed with Egger's regression test (*P* = 0.688) or funnel plot (Supplementary Figure 1). More importantly, sensitivity analyses confirmed that none of the studies affected the overall risk estimate to a significant extend (Supplementary Figure 2). Running a subgroup analysis, country, age adjustment, and sample size were recognized as the sources of heterogeneity. Subgroup analysis also showed the relationship between BMI and mortality in PH patients participating in studies run in US, in which age was adjusted, diabetes was not controlled for, and studies had a shorter duration of follow-up (<5 yrs). Furthermore, Studies which focused on higher sample sizes (≥4,000) revealed more lowered mortality (Tables 2, 3).

**Table 3:** Results of subgroup analysis for body mass index and risk of mortality among pulmonary hypertension patients.

**Group**	**Studies (*n*)**	**ES (95% CI)**	***P***	***P*- within subgroups heterogeneity**	***I*^2^%**	**P-meta-regression**
**Linear dose-response association** **BMI (per 5 kg/m**^**2**^ **increase)**						
**Total**	9	0.83 (0.77, 0.89)	<0.001	<0.001	75.6	
**Country**						
US^a^	6	0.83 (0.79, 0.88)	<0.001	0.075	50	0.169
Non US	3	0.56 (0.27, 1.15)	0.115	<0.001	87	
**Adjustment**						
**Age**						
Yes	4	0.84 (0.76- 0.93)	0.001	<0.001	84.7	0.115
No	2	0.87 (0.73, 1.05)	0.167	0.412	0	
Unclear	3	0.55 (0.29- 1.02)	0.061	0.003	83.2	
**Diabetes**						
Yes	2	0.88 (0.75- 1.03)	0.112	<0.001	92.1	0.086
No	4	0.81 (0.70,0.95)	0.010	0.052	61.2	
Unclear	3	0.55 (0.29- 1.02)	0.061	0.003	83.2	
**Sample size**						
<4,000	7	0.79 (0.68, 0.92)	0.002	<0.001	77	0.674
≥4,000	2	0.83 (0.79- 0.86)	<0.001	0.171	46.7	
**Duration of Follow-up (years)**						
<5	7	0.83 (0.77, 0.89)	<0.001	0.014	62.2	0.425
≥5	2	0.80 (0.55, 1.16)	0.245	0.001	91.6	
**Study qualities**						
High	4	0.80 (0.69, 0.93)	0.005	<0.001	85.9	0.72
Low	5	0.84 (0.76, 0.93)	0.002	0.053	57.2	

a *US, United States*.

**Figure 3:**
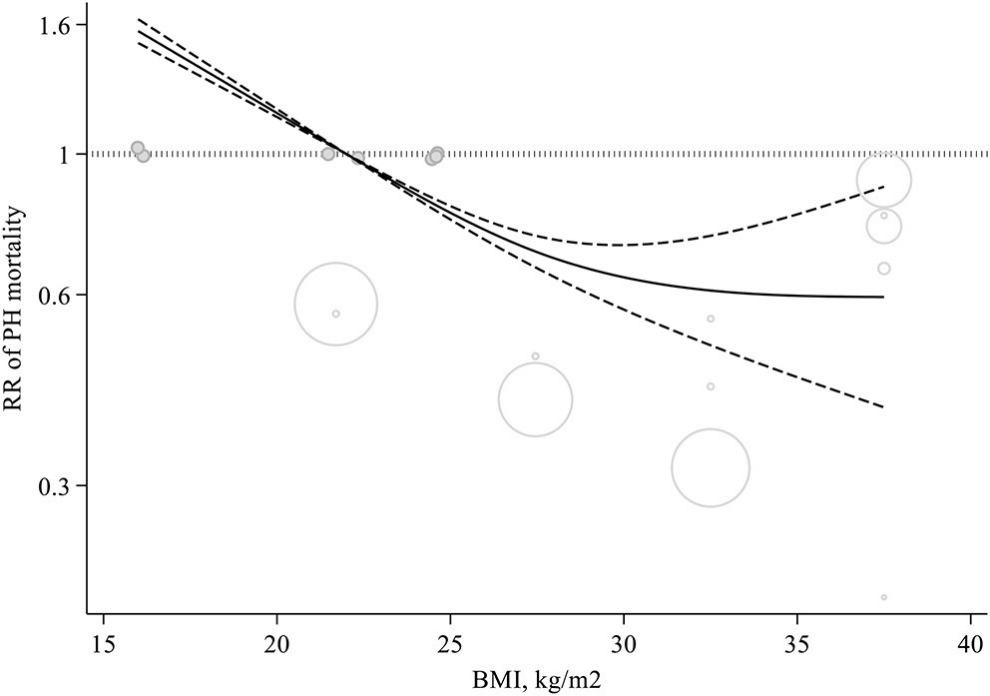
Non-linear dose-response meta-analysis of cohort studies investigating the association between body mass index and mortality among pulmonary hypertension patients (*P* < 0.001).

Some evidence suggested a non-linear dose–response relation between BMI and risk of mortality among those suffering from PH (*P-*non-linearity <0.001, *n* = 7 studies) (Figure 3).

## Discussion

This systematic review and meta-analysis stablished to investigate the relation between BMI and mortality among patients with PH. The most noticeable issue that we came across in our study was that higher BMI was associated to decreased mortality. This entails that the phenomenon known as “obesity paradox” does in fact exist among PH patients. We noticed that 5 kg/m^2^ increment in BMI lowered mortality risk among PH patients to 17%. This relationship was found to be dose-dependent in such a way that mortality risk among the PH patients decreased from BMI 22–23 kg/m^2^ to higher amounts. When BMI reached 32–38 kg/m^2^, the RR was at its lowest.

We realized that among those suffering from PH, higher BMI was associated with decreased mortality. Similarly, in a clinical trial, it was found that when body mass increased by 10 kg/m^2^ the end result was a 10-year mortality decrease (39). Some studies indicated that BMI and long-term mortality were related in an opposite way with respect to hemodialysis as well as some chronic diseases, including; chronic obstructive pulmonary disease (40), peripheral vascular disease (41), coronary artery disease (42, 43). On the other hand, death risk has been confirmed to have a parallel relationship with higher BMI among the general population (44). Furthermore, based on the result of several studies, higher BMI is confirmed to be among the main risk factors for increased risk of PH (7, 12).

Subgroup analysis showed an inverse relationship between BMI and mortality among PH patients in studies conducted in US, those which were adjusted to age, those that not controlled diabetes, as well as those with lower duration of follow-up (<5 years). Obviously, a definite risk factor regarding PH mortality was age. A separate study also claimed that people who aged ≥65 years have higher mortality rates (45). Older obese patients were found to experience more concurrent conditions like diabetes and systemic hypertension (46, 47). Moreover, pulmonary hypertension (PH) is highly common in the United States and affects more than 5 million adults. We also found a growing prevalence in studies adjusted for age and those were carried out in US.

Despite the fact that a persuasive explanation has not yet been proposed for this phenomenon, data suggests that in comparison with non-obese patients, obese ones enjoy a better prognosis, which is in fact due to the abundance of adipose tissue (48). Considering patients suffering from chronic diseases like chronic obstructive pulmonary disease, heart failure, coronary heart disease, and PAH, were found to sustain a higher baseline inflammatory condition, with markers like interleukin-1, interleukin-6, tumor necrosis factor–alpha levels, they undergo higher catabolic burden and cachexia (11). Thus, the extra adipose tissue may ultimately result in a reduction of cytokines in PAH as well which in turn affects the prognosis in a positive way. Additionally, considering that renin-angiotensin system upregulation in late experimental PAH was confirmed to be strongly related to mortality, it is safe to conclude that there is an advantage for those patients who are obese since they are proved to have weakened reaction to renin-angiotensin system (49). Based on the reports of a PH animal model, obesity paradox may, partially, be explained by considering the overexpression of the sympathetic nervous activity which regulates the pulmonary vascular tone in a different way, hence limiting the debilitative impacts of the severity of PH (50). In the same vein, some authors have hypothesized that ischemic preconditioning, from random exposures of chronic hypoxemia, and growing sympathetic nervous activity in obesity, might paradoxically grant a survival advantage (51). Moreover, the overweight and obese groups were found to have a lower pulmonary vascular resistance, although the degree of resistance was similar when indexed to body surface area, therefore no explanation for the differences in the survival observed could be provided based on them (19). Also, obese individuals had higher pulmonary artery wedge pressures, which likely reflects a greater prevalence of concomitant HFpEF, which in turn, leads in a lower trans pulmonary gradient and PVR (19).

This study has some strengths. The prospective design of the included studies was the main strength of the present meta-analysis. Also, linear and non-linear dose-response analysis, provided the most compelling evidence in help us have a quantitative evaluation of relationships. Thanks to this analysis, we could estimate the shape of these possible associations. This relationship was found to be dose-dependent in such a way that mortality risk among the PH patients decreased from BMI 22–23 kg/m^2^ to higher amounts. When BMI reached 32–38 kg/m^2^, the RR was at its lowest.

Limitations of the present study are referral bias and confounding by indication, which might limit the generalizability of the findings to some extent. We studied the association of BMI with mortality among PH patients rather than any general population, which likely reflects referral bias. As mentioned earlier, our data are observational, and therefore no causal inferences can be drawn. The role of residual confounders which result from unmeasured factors such as behavioral and biological ones or biases which inevitably occur in the measurement of covariates, cannot be completely excluded thanks to the observational nature of the included studies. Although there are some limitations using BMI as measure for obesity, it is still the most established and universally accepted indicator for obesity. Moreover, this meta-analysis revealed a noticeable heterogeneity between the studies. Therefore, a subgroup analysis seemed necessary to conduct to discover the cause of heterogeneity; and, we could detect some possible sources in linear analysis. Besides, some of the studies adjusted for factors like diabetes and hypertension which could be either confounders or intermediates in the relationship that could affect the identified associations. Furthermore, most of included studies had low quality which decrease the accuracy of our finding. Finally, we searched only main databases such as PubMed, Scopus and did not search other databases including ISI Web of Knowledge, EMBASE.

In conclusion, higher BMI were confirmed to decrease mortality risk with a dose-response relation among individuals with PH, and the lowest point of the dose-response curve appears to be in the BMI range of 32–38.

It is of outmost importance to stress that this relatively strong association between obesity and favorable survival, which is indeed paradoxical, does not stand for causation and is never meant to tempt people to obtain weight. The current results must be interpreted with caution. The dear reader should know that these results do not (1) confirm a constant causal relationship between weight gain and risk of mortality in PH, (2) support the idea that gaining weight reduces the risk of mortality (as only PH cases were included), and (3) suggest that gaining weight is healthy or leads to disease improvement in PH.

## Data Availability Statement

The original contributions presented in the study are included in the article/Supplementary Material, further inquiries can be directed to the corresponding author/s.

## Author Contributions

CJ wrote the first draft of the manuscript. All authors were contributed to the design of the study, data extraction and analysis, and critically revised and approved the manuscript.

## Conflict of Interest

The authors declare that the research was conducted in the absence of any commercial or financial relationships that could be construed as a potential conflict of interest.

## Publisher's Note

All claims expressed in this article are solely those of the authors and do not necessarily represent those of their affiliated organizations, or those of the publisher, the editors and the reviewers. Any product that may be evaluated in this article, or claim that may be made by its manufacturer, is not guaranteed or endorsed by the publisher.
