# Supplementary material for: The Association of Body Mass Index With Mortality Among Pulmonary Hypertension Patients: A Systematic Review and Meta-Analysis of Cohort Studies
Source: Front Public Health. 2022 May 10;10:761904. doi: 10.3389/fpubh.2022.761904 (PMC9127599; doi:10.3389/fpubh.2022.761904)
Supplement: Supplementary file 1 [file Data_Sheet_1.pdf]

Supplementary Figure 1

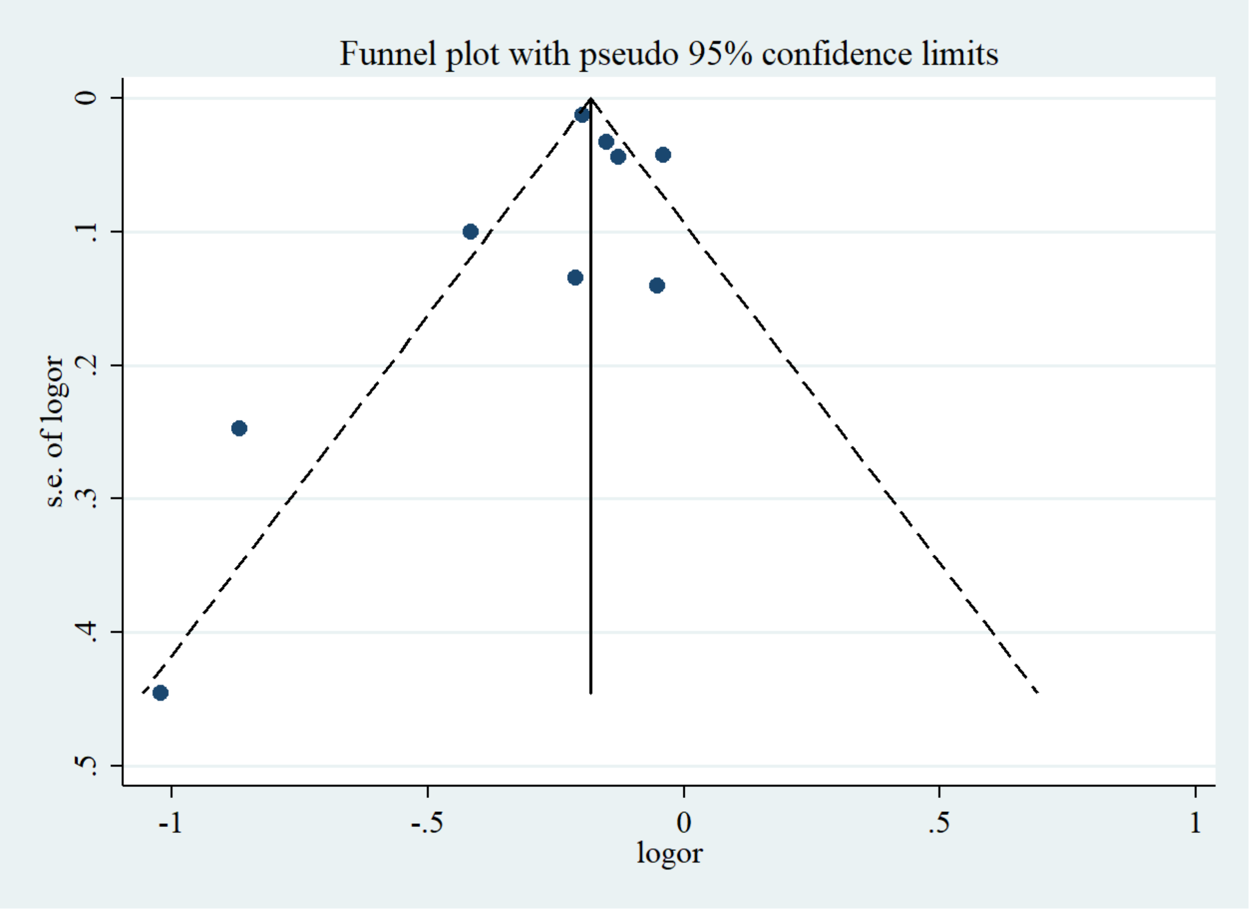

Funnel plot of the risk estimates of nine studies on the association between 5-unit increment in body mass index and risk of mortality among pulmonary hypertension patients. Each dot represents one study.

Supplementary Figure 2

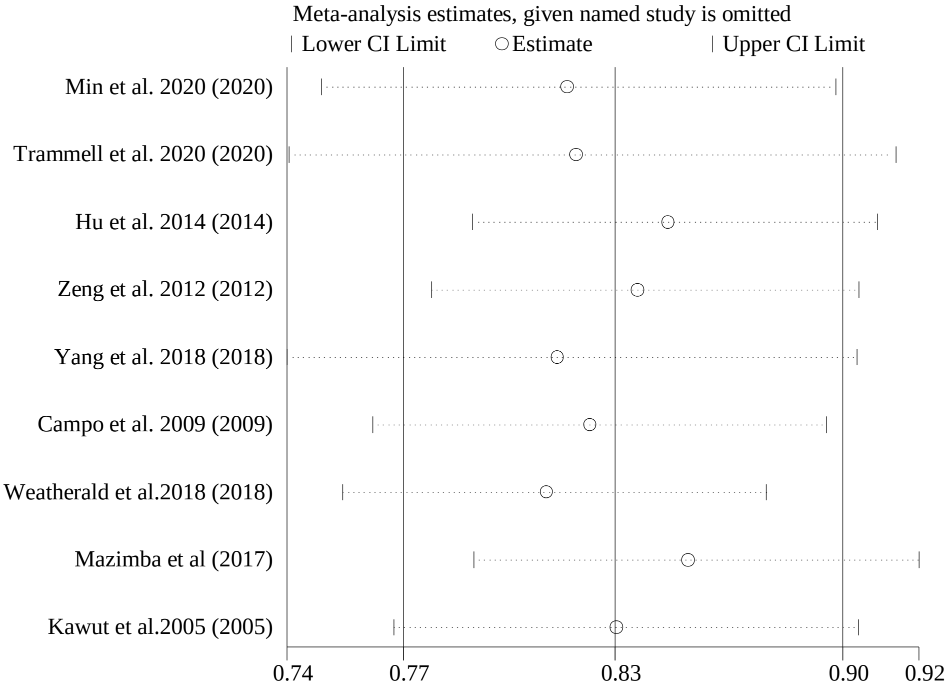

Sensitivity analysis of the association between 5-unit increment in body mass index and risk of mortality among pulmonary hypertension patients. CI, confidence interval
